# Fasting Decreases the Content of D-Chiroinositol in Human Skeletal Muscle

**DOI:** 10.1080/15604280214280

**Published:** 2002

**Authors:** Pavel N. Shashkin, Laura C. Huang, Joseph Larner, George E. Vandenhoff, Abram Katz

**Affiliations:** 1 Department of Internal Medicine University of Virginia Charlottesville Charlottesville Virginia USA; 2 Department of Pharmacology University of Virginia Charlottesville Charlottesville Virginia USA; 3 Department of Physiology and Pharmacology Karolinska Institutet Stockholm 171 77 Sweden

## Abstract

Two classes of inositol phosphoglycans have been implicated as second messengers of insulin, one that activates pyruvate dehydrogenase and contains D-chiroinositol, and one that inhibits cyclic AMP–dependent protein kinase and contains myoinositol. We examined the effects of a 3-day fast on muscle contents of inositols in healthy humans. An oral glucose tolerance test was performed and a biopsy was obtained from the quadriceps femoris muscle after an overnight fast and after a 72-hour fast. The 72-hour fast significantly increased plasma glucose (1.5- to 2-fold) and insulin (2- to 4-fold) after glucose ingestion versus the values after the overnight fast, indicating the manifestation of peripheral insulin resistance. The 72-hour fast resulted in an ∼20% decrease in the muscle content of D-chiroinositol (*P* < 0.02), but no change in the myoinositol content. These data demonstrate that fasting specifically decreases the muscle content of D-chiroinositol in human muscle and this may contribute to the finding that insulin-mediated activation of pyruvate dehydrogenase is attenuated after short-term starvation.


*Int. Jnl. Experimental Diab. Res.*, 3:163–169, 2002
Copyright © 2002 Taylor & Francis
1560-4284/02 $12.00 + .00
DOI: 10.1080/15604280290013865

### Fasting Decreases the Content of D-Chiroinositol in Human Skeletal Muscle

**Pavel N. Shashkin,[1] Laura C. Huang,[2] Joseph Larner,[2] George E. Vandenhoff,[2] and Abram Katz[3]**

[1]*Department of Internal Medicine, University of Virginia Charlottesville, Charlottesville, Virginia, USA*
[2]*Department of Pharmacology, University of Virginia Charlottesville, Charlottesville, Virginia, USA*
[3]*Department of Physiology and Pharmacology, Karolinska Institutet, Stockholm, Sweden*

**Two classes of inositol phosphoglycans have been implicated as second messengers of insulin, one that activates pyruvate dehydrogenase and contains D-chiroinositol, and one that inhibits cyclic AMP–dependent protein kinase and contains myoinositol. We examined the effects of a 3-day fast on muscle contents of inositols in healthy humans. An oral glucose tolerance test was performed and a biopsy was obtained from the quadriceps femoris muscle after an overnight fast and after a 72-hour fast. The 72-hour fast significantly increased plasma glucose (1.5- to 2-fold) and insulin (2- to 4-fold) after glucose ingestion versus the values after the overnight fast, indicating the manifestation of peripheral insulin resistance. The 72-hour fast resulted in an ∼20% decrease in the muscle content of D-chiroinositol ($P < 0.02$), but no change in the myoinositol content. These data demonstrate that fasting specifically decreases the muscle content of D-chiroinositol in human muscle and this may contribute to the finding that insulin-mediated activation of pyruvate dehydrogenase is attenuated after short-term starvation.**

**Keywords** D-Chiroinositol; Fasting; Glucose; Insulin; Myoinositol; Oral Glucose Tolerance

Larner and coworkers first proposed the existence of a putative mediator of insulin action [1]. This idea was based on the dissociation of several actions of insulin, which suggested that a second step after insulin binding must involve separate pathways. Subsequently, several insulin mediators were isolated from various mammalian tissues [2, 3]. In general, the isolated mediators consist of inositols linked to hexosamines, hexoses, organic phosphorus, and ethanolamine and are therefore termed inositol phosphoglycans (IPGs) [4–6]. The isolated IPGs mimic a number of insulin effects in vivo, on isolated cells and on purified enzymes [7, 8]. Specifically, the IPG that contains myoinositol inhibits purified cyclic AMP–dependent protein kinase, and the IPG that contains D-chiroinositol activates pyruvate dehydrogenase phosphatase (PDH phosphatase) [9]. In this context, it is noteworthy that insulin-resistant, non–insulin dependent diabetics exhibit relatively low levels of D-chiroinositol and low bioactivity of the IPG that contains D-chiroinositol isolated from several tissues [10, 11]. Similarly, the increase in the bioactivity of the IPG that contains D-chiroinositol, as well as the D-chiroinositol content of the isolated IPG from diabetics, in response to acute increases in plasma insulin is also smaller than corresponding values for control subjects [11, 12].

In addition to diabetes, short-term starvation is also associated with insulin resistance in humans. Specifically, short-term starvation results in decreased basal and insulin-mediated muscle glucose uptake and carbohydrate oxidation [13–15], which may be a consequence of diminished D-chiroinositol availability. The purpose of the present study, therefore, was to determine the effects of a 3-day fast on the muscle contents of inositols in healthy humans.

Received 28 October 2001; accepted 21 February 2002.
This research was supported by grants from Insmed Pharmaceuticals, Gamla Tjänarinnor Foundation, and Karolinska Institutet. The authors thank Drs. Barbara Hansen and Heidi Ortmeyer for useful discussions and help during the course of the study.
Address correspondence to Abram Katz, Department of Physiology and Pharmacology, Karolinska Institutet, 171 77 Stockholm, Sweden. E-mail: abram.katz@fyfa.ki.se

### MATERIALS AND METHODS

### Subjects

A total of 12 healthy volunteers participated in the study (see Table 1). The study protocol was reviewed and approved by the ethics committee of Karolinska Hospital and informed written consent was given by all participants.

### Protocol

Subjects reported to the laboratory after an overnight (10-hour) fast and assumed the supine position. A local anesthetic (carbocain, 5 mg/ml) was administered and an incision was made over the lateral aspect of the quadriceps femoris muscle of one thigh. After ~15 minutes, a biopsy was taken. Thereafter, a plastic catheter was inserted into an antecubital vein for blood sampling. A basal blood sample was drawn and the subjects ingested 75 g of glucose dissolved in 375 mL of water. Additional blood samples were drawn at 60 and 120 minutes after glucose ingestion. The subjects returned to the laboratory ~72 hours later and the protocol described above was repeated, with the exception that muscle sampling was from the contralateral leg. During the 72-hour interim, 8 subjects fasted, but were allowed to drink water ad libitum. The remaining 4 control subjects ate and drank normally during the interim, but also fasted overnight prior to the second trial.

### Analytical

Plasma glucose was measured with the glucose oxidase technique using a glucose analyzer (Beckman Instruments, Fullerton, CA, USA). Plasma insulin was measured by radioimmunoassay and plasma $\beta$-hyroxybutyrate with an enzymatic fluorometric technique [16].

Biopsies were quick-frozen in liquid nitrogen. Biopsies were lyophilized, dissected free from nonmuscle constituents, powdered, and thoroughly mixed, aliquoted, and stored at −80°C. For analysis of glycogen, an aliquot of muscle powder was digested in hot 1 M KOH, neutralized, hydrolyzed enzymatically, and analyzed for free glucose [17]. Another aliquot was homogenized, centrifuged, diluted, and analyzed for glycogen synthase and phosphorylase activities using filter paper techniques [18, 19] as described elsewhere [20]. Glycogen synthase fractional activity is the activity measured in the presence of 0.17 mM glucose-6-phosphate divided by the activity measured at saturating glucose-6-phosphate (7.2 mM). Phosphorylase fractional activity is the activity measured in the absence divided by the activity measured in the presence of 3.3 mM AMP. Protein was measured in the enzyme extracts with the BioRad protein assay.

For analysis of inositols, aliquots of powder were suspended in 6 N HCl. The tubes were sealed and heated at 100°C for 48 hours. Thereafter, the extracts were diluted in distilled water and lyophylized. The latter step was repeated 2 more times. Analyses of chiro- and myoinositols were performed by radioimmunoassay using specific antibodies and iodinated tracers developed in this laboratory (manuscript in preparation). Myoinositol was from Sigma (St. Louis, MO, USA). D-Chiroinositol was obtained as a hydrolysis product of pinetol, originally extracted from pine sawdust. Polyclonal antisera to chiro- and myoinositol were generated in rabbits, using as immunogens the corresponding monosuccinyl- and pentaacetylinositol, conjugated via the succinate group directly to bovine serum albumin (Sigma RIA grade). Similarly configured monosuccinyl- and pentaacetylinositol conjugated to tyramine were used as the starting point for chloramine T iodination and subsequent purification of the iodinated tracers by reverse phase C18 high-performance liquid chromatography (HPLC). The assay required complete acetylation (hexaacetylation) of inositol species in standard and samples before assay; nonacetylated inositols did not cross-react with their respective antibodies. Complete acetylation was achieved as follows. One hundred microliters of standard or sample (in a 1:20 dilution) in 1 mg/ml urea and 0.1% sodium azide were dried in a 12 × 75-mm polypropylene test tube, to which were then added 100 $\mu$l of an acetic anhydride (32.5%)/pyridine (65%)/triethylamine (2.5%) mix, vortexed thoroughly, and allowed to incubate overnight at room temperature. The next day, the incubates were dried in a vacuum oven, with special care to remove the last traces of pyridine. For assay, 100 $\mu$l of sample or

**TABLE 1**
Subject characteristics

| Group | M/F | Age (year) | Height (cm) | BMI (kg/m$^2$) | Pre-weight (kg) | Post-weight (kg) |
|---|---|---|---|---|---|---|
| Fasted | 5/3 | $32 \pm 2$ | $174 \pm 3$ | $23.5 \pm 0.4$ | $71.1 \pm 3.4$ | $67.9 \pm 3.2^{***}$ |
| Control | 3/1 | $27 \pm 3$ | $178 \pm 4$ | $22.1 \pm 1.8$ | $69.7 \pm 7.3$ | $69.4 \pm 7.1$ |

*Note.* Values are mean ± SE for 8 (Fasted) and 4 (Control) subjects, respectively. Pre, after overnight fast; Post, after 72-hour fast (Fasted group) or overnight fast on a second occasion (Control). $^{***}P < 0.05$ versus Pre.

standard were added to 12 × 75-mm polypropylene tubes, together with 100 $\mu$l of antiserum (1:1000 dilution for chiroinositol antiserum; 1:2500 to myoinositol antiserum) and 100 $\mu$l of iodinated tracer. After overnight incubation at room temperature, bound inositols were precipitated using polyethylene glycol (PEG)-assisted double-antibody precipitation. One hundred microliters of a 1/100 dilution of goat anti-rabbit antiserum (Sigma) and (to myoinositol assay tubes only) 100 $\mu$l of a 1/1000 dilution of normal rabbit serum were added to each tube and incubated for 1 hour at room temperature. Then 1.25 ml of ice-cold 12% PEG (average MW = 8000; Sigma) were added to each tube, which was then vortexed and incubated an additional 1.5 hour in an ice bath. Tubes were spun at 4000 rpm for 1 hour before pouring off supernatant and counting pellets. Concentrations were calculated off a logit-log transformation of the bound counts per minute (cpm) data.

### Statistics

Statistically significant differences ($P < 0.05$) between means were determined with the paired $t$ test. Values are presented as mean ± SE unless otherwise indicated.

### RESULTS

The 3-day fast resulted in an almost 5% decrease in body weight (Table 1) and basal concentrations of plasma glucose and insulin that amounted to, respectively, ~70% and 50% of the overnight fasted values (Figure 1, *right panels*). These results are similar to an earlier report using the same fasting protocol [13]. Additionally, the 3-day fast increased basal plasma $\beta$-hydroxybutyrate concentrations almost 40-fold (overnight fast = 51 ± 13 $\mu$M; 3-day fast = 1973 ± 345 $\mu$M; $P < 0.001$). The increases in plasma glucose and insulin after the oral

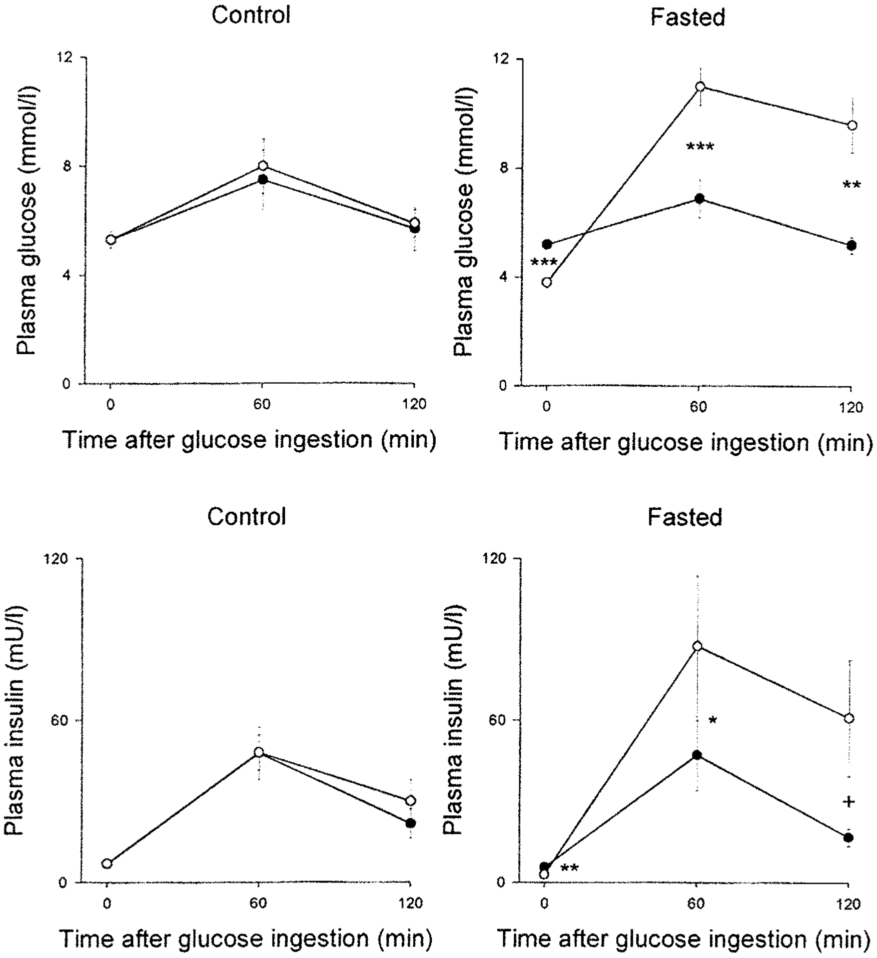

**FIGURE 1**
Plasma glucose and insulin responses to glucose ingestion. Values are mean ± SE for 8 (Fasted) and 4 (Control) subjects, respectively. Subjects were studied on the first occasion after an overnight fast (filled circles) and on the second occasion (unfilled circles) after a 72-hour fast (Fasted) or again after an overnight fast (Control). $^{*}P < 0.05$; $^{**}P < 0.01$; $^{***}P < 0.001$; $^{+}P = 0.07$ versus first test.

**TABLE 2**
Effects of fasting on muscle glycogen and enzyme activities

| | Control | | Fasted | |
|---|---|---|---|---|
| | Pre | Post | Pre | Post |
| Glycogen | 333 ± 25 | 278 ± 50 | 402 ± 11 | 336 ± 38 |
| $GS_H$ | 3.47 ± 0.43 | 4.12 ± 0.25 | 3.81 ± 0.39 | 3.46 ± 0.33* |
| GSF | 0.32 ± 0.05 | 0.35 ± 0.04 | 0.35 ± 0.03 | 0.30 ± 0.02 |
| Phos | 209 ± 7 | 203 ± 29 | 185 ± 17 | 196 ± 12 |
| PF | 0.19 ± 0.02 | 0.20 ± 0.03 | 0.21 ± 0.04 | 0.22 ± 0.02 |
| Protein | 181 ± 6 | 192 ± 21 | 183 ± 14 | 187 ± 12 |

*Note.* Values are mean ± SE for 8 (Fasted) and 4 (Control) subjects, respectively. Glycogen is expressed as mmol glucosyl units/kg dry muscle, whereas glycogen synthase (+7.2 mM glucose 6-P, $GS_H$) and phosphorylase (+3.3 mM AMP, Phos) are expressed as mmol/min/kg dry muscle, and protein g/kg dry muscle. $^*P < 0.05$ versus Pre.

glucose-tolerance test after the 3-day fast were significantly greater than those observed after the overnight fast. In the control group, plasma concentrations of glucose and insulin, as well as $\beta$-hyroxybutyrate (data not shown), were comparable on the 2 occasions, both before and after glucose ingestion (Figure 1, *left panels*).

The 3-day fast decreased glycogen synthase activity (+7.2 mM glucose-6-phosphate) in muscle by about 10% (Table 2), which is similar to previous findings [13]. Concomitantly, muscle glycogen content and glycogen synthase fractional activity tended to decrease after the 3-day fast, but the decreases did not reach statistical significance. Neither muscle phosphorylase activity (+3.3 mM AMP), phosphorylase fractional activity, nor protein content was affected by the 3-day fast. No differences in any of these variables were noted in the control group.

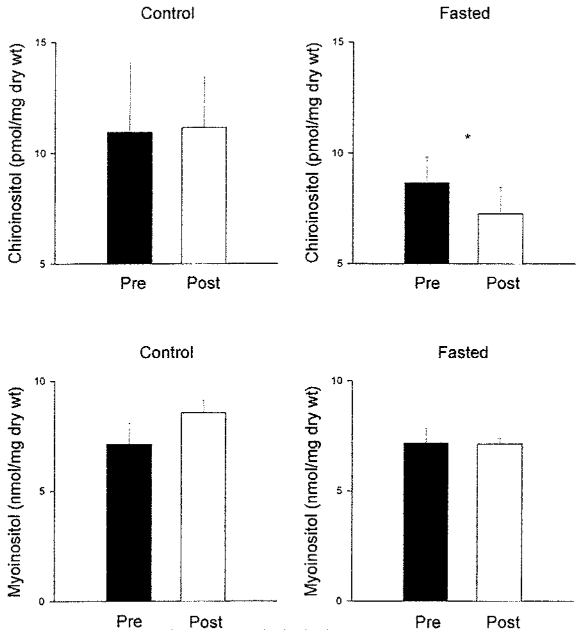

**FIGURE 2**
Muscle inositol contents. Values are means ± SE for 6 to 7 (Fasted) or 3 to 4 (Control) subjects. Pre and Post denote before and after the 72-hour periods, respectively. $^*P < 0.02$.

The 3-day fast resulted in a significant decrease in the muscle content of D-chiroinositol (from 8.64 ± 1.19 to 7.25 ± 1.22 pmol/mg dry muscle), whereas the myoinositol content was unaffected (Pre = 7.18 ± 0.67, Post = 7.14 ± 0.25 nmol/mg dry muscle) (Figure 2, *right panels*). The decrease in D-chiroinositol averaged 16% and similar results were obtained if the values were adjusted for protein (19% decrease, $P = 0.06$) or phosphorylase (22% decrease, $P < 0.05$). There were no significant differences in the inositols in the control group.

### DISCUSSION

The major finding in this study is that short-term starvation significantly decreased the content of D-chiroinositol in human skeletal muscle. The decrease was associated with the manifestation of peripheral insulin resistance, as indicated by the exaggerated increases in plasma glucose and insulin following glucose ingestion, as well as the decrease in glucose disposal and carbohydrate oxidation during euglycemic hyperinsulinemia [13].

The present results raise the question regarding the relationship between the decreased muscle D-chiroinositol content and insulin resistance. Published studies from several laboratories have already established a clear relationship between D-chiroinositol and insulin resistance. Thus in the Rhesus monkey, a linear relationship between the 24-hour urinary chiroinositol excretion rate and insulin resistance was demonstrated by 5

independent methods, including glucose disposal rates during euglycemic hyperinsulinemia, serum glucose disappearance rates after intravenous glucose injection, and muscle and fat biopsy determinations of glycogen synthase and phosphorylase activity states [21]. Japanese subjects with normal glucose tolerance, impaired glucose tolerance, and type II diabetes were compared with regard to 24-hour urinary chiroinositol excretion and insulin sensitivity. Again, a linear relationship was observed between 24-hour urinary chiroinositol excretion and insulin sensitivity, with normals having the highest excretion rates, the impaired glucose tolerance patients having decreased excretion and the diabetics the lowest excretion rates [22]. Previous studies have also demonstrated that non–insulin dependent diabetes is associated with decreases in the D-chiroinositol content of IPGs isolated from skeletal muscle [11]. Concomitantly, the bioactivity of the IPG that contains D-chiroinositol, isolated from muscle and other body fluids, is also diminished in non–insulin dependent diabetes [10]. Moreover, the increase in the bioactivity of the D-chiroinositol–containing IPG in response to acute increases in insulin is diminished in non–insulin dependent diabetic patients, who are insulin resistant [11, 12]. Thus, the current results that simply associate decreased D-chiroinositol content in muscle with insulin resistance are in keeping with a body of published data that more directly relate decreased D-chiroinositol in urine and tissue with insulin resistance. This relationship is further supported by the findings that administration of D-chiroinositol or pinitol (3-*O*-methyl-D-chironinositol) decreases elevated blood glucose and improves insulin resistance in animal models of type II diabetes [23–25] and humans with impaired glucose tolerance, type II diabetes, and polycystic ovary disease [26–28]. In view of the above, the current findings could be supplemented by measurements of IPG release following glucose ingestion, as well as assessment of urinary inositol excretion before and after short-term starvation.

PDH is activated via dephosphorylation by PDH phosphatases and these phosphatases are activated in vitro by the D-chiroinositol–containing IPG [9, 29]. Others have provided evidence that mediators of insulin can also activate PDH in isolated mitochondria [30, 31]. Short-term starvation decreases the plasma insulin concentration (see above) and the activity of the active (dephosphorylated) form of pyruvate dehydrogenase in insulin-sensitive tissues [32–34]. Moreover, fasting also decreases the PDH-activating capacity of an activator isolated from hearts [35] of insulin-treated rats. Similarly, fasting also decreases the PDH-activating capacity of an activator isolated from insulin-treated plasma membranes isolated from rat hepatocytes [31], as well as from insulin-treated rat liver particulate fractions [36]. The current findings provide a potential mechanism for the negative effects of fasting on insulin-mediated activation of PDH, namely, that there is insufficient D-chiroinositol to generate the fully active IPG.

The mechanism for the decrease in the content of D-chiroinositol in muscle after a 3-day fast is not known. One possibility is that the decrease in D-chiroinositol is due to lack of dietary intake. On the other hand, the myoinositol content was not decreased after the 3-day fast. Alternatively, the decrease in D-chiroinositol may be related to a decreased capacity to convert myoinositol to D-chiroinositol. Myoinositol is converted to D-chiroinositol in nonmammalian tissue by an oxidoreductive epimerization of the C3 hydroxyl of myoinositol [37]. However, this enzymatic reaction has not been shown to produce chiroinositol in animal tissue [38]. Nevertheless, acute insulin administration in diabetics increases plasma D-chiroinositol concentrations by almost 10-fold and this is associated with a significant decrease in the plasma myoinositol concentration [39]. Further, administration of [$^3$H]myoinositol to rats results in significant production of [$^3$H]chiroinositol in insulin-sensitive tissues [40], and administration of insulin to rat fibroblasts expressing the human insulin receptor results in significant conversion of [$^3$H]myoinositol to [$^3$H]chiroinositol phospholipids within 15 minutes. These results support the idea that short-term starvation diminishes the conversion of myoinositol to D-chiroinositol, thereby explaining the decrease in D-chiroinositol content after the 3-day fast.

In conclusion, a 3-day fast results in a decrease in D-chiroinositol content in human skeletal muscle. This decrease may be responsible for the attenuation of some of insulin's actions.

### REFERENCES

[1] Larner, J., Huang, L. C., Brooker, G., Murad, F., and Miller, T. B., Jr. (1974) Inhibitor of protein kinase formed in insulin treated muscle. *Fed. Proc.,* **33,** 261.

[2] Larner, J., Galasko, G., Cheng, K., DePaoli-Roach, A. A., Huang, L., Daggy, P., and Kellogg, J. (1979) Generation by insulin of a chemical mediator that controls protein phosphorylation and dephosphorylation. *Science,* **206,** 1408–1410.

[3] Saltiel, A., Jacobs, S., Siegel, M., and Cuatrecasas, P. (1981) Insulin stimulates the release from liver plasma membranes of a chemical modulator of pyruvate dehydrogenase. *Biochem. Biophys. Res. Commun.,* **102,** 1041–1047.

[4] Larner, J. (1988) Insulin-signaling mechanisms. Lessons from the old testament of glycogen metabolism and the new testament of molecular biology. *Diabetes,* **37,** 262–275.

[5] Mato, J. M., Kelly, K. L., Abler, A., and Jarett, L. (1987) Identification of a novel insulin-sensitive glycophospholipid from H35 hepatoma cells. *J. Biol. Chem.,* **262,** 2131–2137.

[6] Saltiel, A. R., Fox, J. A., Sherline, P., and Cuatrecasas, P. (1986) Insulin-stimulated hydrolysis of a novel glycolipid generates modulators of cAMP phosphodiesterase. *Science,* **233,** 967–972.

[7] Larner, J., and Huang, L. C. (1999) Identification of a novel inositol glycan signaling pathway with significant therapeutic relevance to insulin resistance: An insulin signaling model using both tyrosine kinase and G-proteins. *Diabetes Rev.,* **7,** 217–231.

[8] Romero, G., and Larner, J. (1993) Insulin mediators and the mechanism of insulin action. *Adv. Pharmacol.,* **24,** 21–50.

[9] Larner, J., Huang, L. C., Suzuki, S., Tang, G., Zhang, C., Schwartz, C. F., Romero, G., Luttrell, L., and Kennington, A. S. (1989) Insulin mediators and the control of pyruvate dehydrogenase complex. *Ann. N. Y. Acad. Sci.,* **573,** 297–305.

[10] Asplin, I., Galasko, G., and Larner, J. (1993) Chiro-inositol deficiency and insulin resistance: A comparison of the chiro-inos. *Proc. Natl. Acad. Sci. USA,* **90,** 5924–5928.

[11] Kennington, A. S., Hill, C. R., Craig, J., Bogardus, C., Raz, I., Ortmeyer, H. K., Hansen, B. C., Romero, G., and Larner, J. (1990) Low urinary chiro-inositol excretion in non-insulin-dependent diabetes mellitus. *N. Engl. J. Med.,* **323,** 373–378.

[12] Shashkin, P., Shashkina, E., Fernqvist-Forbes, E., Zhou, Y. P., Grill, V., and Katz, A. (1997) Insulin mediators in man: Effects of glucose ingestion and insulin resistance. *Diabetologia,* **40,** 557–563.

[13] Castillo, C. E., Katz, A., Spencer, M. K., Yan, Z., and Nyomba, B. L. (1991) Fasting inhibits insulin-mediated glycolysis and anaplerosis in human skeletal muscle. *Am. J. Physiol.,* **261,** E598–E605.

[14] Owen, O. E., and Reichard, G. A. (1971) Human forearm metabolism during progressive starvation. *J. Clin. Invest.,* **50,** 1536–1545.

[15] Pozefsky, T., Tancredi, R. G., Moxley, R. T., Dupre, J., and Tobin, J. D. (1976) Metabolism of forearm tissues in man. Studies with glucagon. *Diabetes,* **25,** 128–135.

[16] Williamson, D. H., Mellanby, J., and Krebs, H. A. (1962) Enzymatic determination of D(−)-$\beta$-hydroxy-butyric acid and acetic acid in blood. *Biochem. J.,* **82,** 90–96.

[17] Harris, R. C., Hultman, E., and Nordesjo, L. O. (1974) Glycogen, glycolytic intermediates and high-energy phosphates determined in biopsy samples of musculus quadriceps femoris of man at rest. Methods and variance of values. *Scand. J. Clin. Lab. Invest.,* **33,** 109–120.

[18] Tan, A. W., and Nuttall, F. Q. (1975) Characteristics of the dephosphorylated form of phosphorylase purified from rat liver and measurement of its activity in crude liver preparations. *Biochim. Biophys. Acta,* **410,** 45–60.

[19] Thomas, J. A., Schlender, K. K., and Larner, J. (1968) A rapid filter paper assay for UDPglucose-glycogen glucosyltransferase, including an improved biosynthesis of UDP-14C-glucose. *Anal. Biochem.,* **25,** 486–499.

[20] Jiao, Y., Shashkina, E., Shashkin, P., Hansson, A., and Katz, A. (1999) Manganese sulfate-dependent glycosylation of endogenous glycoproteins in human skeletal muscle is catalyzed by a nonglucose 6-P-dependent glycogen synthase and not glycogenin. *Biochim. Biophys. Acta,* **1427,** 1–12.

[21] Ortmeyer, H. K., Bodkin, N. L., Lilley, K., Larner, J., and Hansen, B. C. (1993) Chiroinositol deficiency and insulin resistance. I. Urinary excretion rate of chiroinositol is directly associated with insulin resistance in spontaneously diabetic rhesus monkeys. *Endocrinology,* **132,** 640–645.

[22] Suzuki, S., Kawasaki, H., Satoh, Y., Ohtomo, M., Hirai, M., Hirai, A., Hirai, S., Onoda, M., Matsumoto, M., Hinokio, Y., Akai, H., Craig, J., Larner, J., and Toyota, J. (1994) Urinary chiro-inositol excretion is an index marker of insulin sensitivity in Japanese type II diabetes. *Diabetes Care,* **17,** 1465–1468.

[23] Bates, S. H., Jones, R. B., and Bailey, C. J. (2000) Insulin-like effect of pinitol. *Br. J. Pharmacol.,* **130,** 1944–1948.

[24] Fonteles, M. C., Almeida, M. Q., and Larner, J. (2000) Antihyperglycemic effects of 3-*O*-methyl-D-chiro-inositol and D-chiro-inositol associated with manganese in streptozotocin diabetic rats. *Horm. Metab. Res.,* **32,** 129–132.

[25] Ortmeyer, H. K., Huang, L. C., Zhang, L., Hansen, B. C., and Larner, J. (1993) Chiroinositol deficiency and insulin resistance. II. Acute effects of D-chiroinositol administration in streptozotocin-diabetic rats, normal rats given a glucose load, and spontaneously insulin-resistant rhesus monkeys. *Endocrinology,* **132,** 646–651.

[26] Larner, J. D-Chiro-inositol and insulin resistance. An allosteric point of view. *Int. J. Exp. Diab. Res.,* **3,** xxx–xxx.

[27] Larner, J., Allan, G., Kessler, C., Reamer, P., Gunn, R., and Huang, L. C. (1998) Phosphoinositol glycan derived mediators and insulin resistance. Prospects for diagnosis and therapy. *J. Basic Clin. Physiol. Pharmacol.,* **9,** 127–137.

[28] Nestler, J. E., Jakubowicz, D. J., Reamer, P., Gunn, R. D., and Allan, G. (1999) Ovulatory and metabolic effects of D-chiro-inositol in the polycystic ovary syndrome. *N. Engl. J. Med.,* **340,** 1314–1320.

[29] Lilley, K., Zhang, C., Villar-Palasi, C., Larner, J., and Huang, L. (1992) Insulin mediator stimulation of pyruvate dehydrogenase phosphatases. *Arch. Biochem. Biophys.,* **296,** 170–174.

[30] Kiechle, F. L., Jarett, L., Popp, D. A., and Kotagal, N. (1980) Isolation from rat adipocytes of a chemical mediator for insulin activation of pyruvate dehydrogenase. *Diabetes,* **29,** 852–855.

[31] Trowbridge, M., Sussman, A., Ferguson, L., Draznin, B., Neufeld, N., Begum, N., Tepperman, H., and Tepperman, J. (1984) Mechanisms of the fasting-induced dissociation of insulin binding from its action in isolated rat hepatocytes. *Mol. Cell Biochem.,* **62,** 25–36.

[32] Hagg, S. A., Taylor, S. I., and Ruberman, N. B. (1976) Glucose metabolism in perfused skeletal muscle. Pyruvate dehydrogenase activity in starvation, diabetes and exercise. *Biochem. J.,* **158,** 203–210.

[33] Kruszynska, Y. T., and McCormack, J. G. (1989) Effect of nutritional status on insulin sensitivity in vivo and tissue enzyme activities in the rat. *Biochem. J.,* **258,** 699–707.

[34] Wieland, O., Siess, E., Schulze-Wethmar, F. H., von Funcke, H. G., and Winton, B. (1971) Active and inactive forms of pyruvate dehydrogenase in rat heart and kidney: Effect of diabetes, fasting, and refeeding on pyruvate dehydrogenase interconversion. *Arch. Biochem. Biophys.,* **143,** 593–601.

[35] Macaulay, S. L., Macaulay, J. O., and Jarett, L. (1985) Insulin stimulates generation of intracellular mediators in rat heart. *Arch. Biochem. Biophys.,* **241,** 432–437.

[36] Amatruda, J. M., and Chang, C. L. (1983) Insulin resistance in the liver in fasting and diabetes mellitus: The failure of insulin to stimulate the release of a chemical modulator of pyruvate dehydrogenase. *Biochem. Biophys. Res. Commun.,* **112,** 35–41.

[37] Hipps, P. P., Sehgal, R. K., Holland, W. H., and Sherman, W. R. (1973) Identification and partial characterization of inositol: $NAD^+$ epimerase and inosose: NAD(P)H reductase from the fat body of the American cockroach, Periplaneta americana L. *Biochemistry,* **12,** 4507–4512.

[38] Hipps, P. P., Ackermann, K. E., and Sherman, W. R. (1982) Inositol epimerase–inosose reductase from bovine brain. *Methods Enzymol.,* **89**(Pt D), 593–598.

[39] Ostlund, R. E., McGill, J. B., Herskowitz, I., Kipnis, D. M., Santiago, J. V., and Sherman, W. R. (1993) D-Chiro-inositol metabolism in diabetes mellitus. *Proc. Natl. Acad. Sci. USA,* **90,** 9988–9992.

[40] Pak, Y., Huang, L. C., Lilley, K. J., and Larner, J. (1992) In vivo conversion of [3H]myoinositol to [3H]chiroinositol in rat tissues. *J. Biol. Chem.,* **267,** 16904–16910.

